# Masters of Disguise: Antigenic Variation and the VSG Coat in *Trypanosoma brucei*

**DOI:** 10.1371/journal.ppat.1005784

**Published:** 2016-09-01

**Authors:** Monica R. Mugnier, C. Erec Stebbins, F. Nina Papavasiliou

**Affiliations:** 1 The Rockefeller University, Laboratory of Lymphocyte Biology, New York, New York, United States of America; 2 The Rockefeller University, Laboratory of Structural Microbiology, New York, New York, United States of America; University of Wisconsin Medical School, UNITED STATES

## What Is VSG?

**Fig 1:**
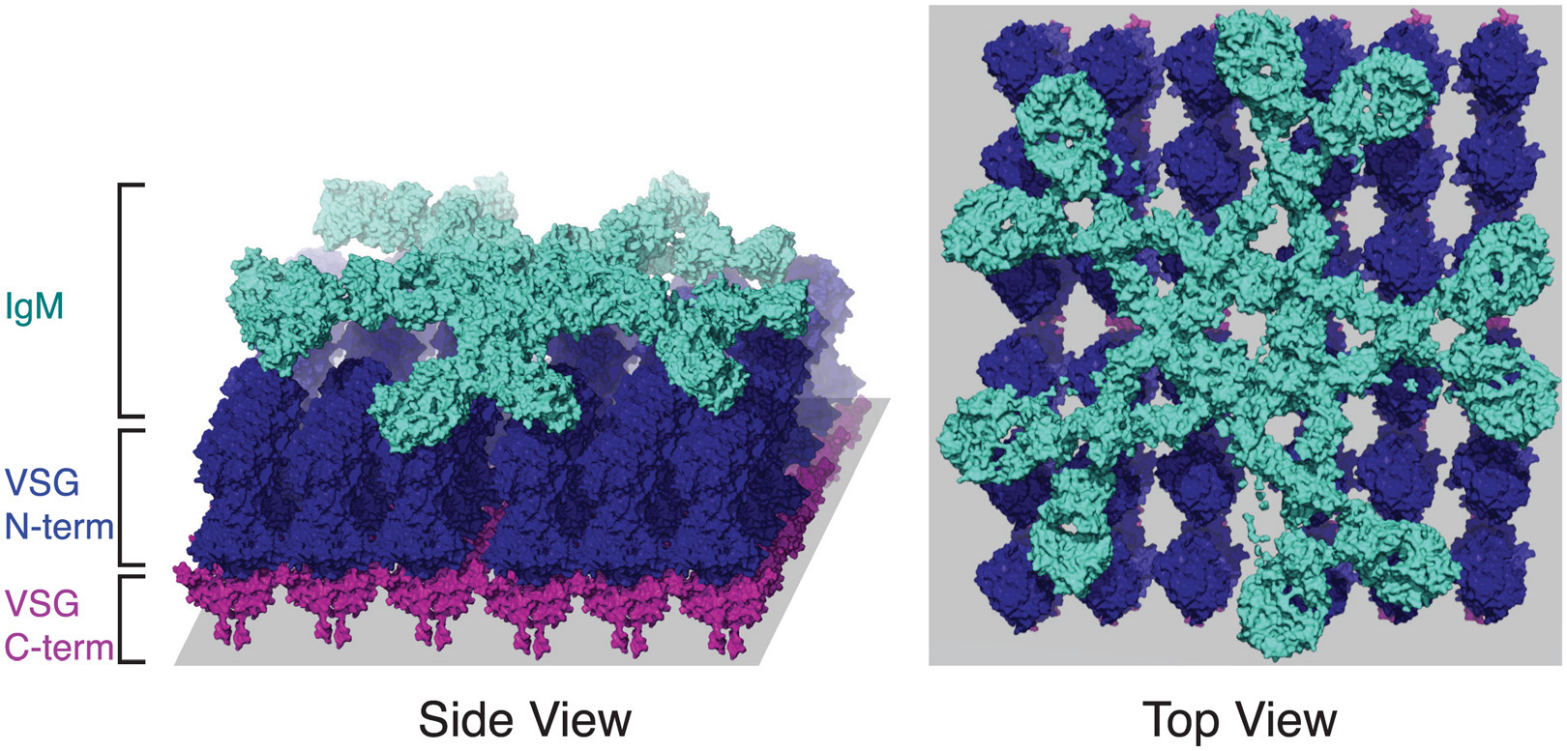
A model of the VSG–antibody interaction. A hypothetical model of immunoglobulin M (IgM) antibody (teal) binding to VSG (pink and blue). The precise arrangement of VSG on the cell membrane (gray) is unknown, but the packing of VSG on the cell membrane is known to be extremely dense. It is unknown whether IgM binds VSG in this particular configuration, but the dense packing of VSG may nevertheless affect the accessibility of antibody to the C-terminus (pink), as illustrated here. The multimeric IgM is also likely to interact with more than one VSG on the cell surface, resulting in the depicted “staple” conformation. The figure was produced by combining (1) the pentameric C-alpha model of IgM (PDB ID 2RCJ), (2) modeling the remainder of the chains using the FG-MD Server [8] over (3) a planar array of manually positioned VSG N-terminal and C-terminal domains based on the crystal structures of VSG221 (PDB ID 1VSG, 1XU6).

VSG stands for variant surface glycoprotein, the major surface component of the protozoan parasite *Trypanosoma brucei* while it exists in the blood and tissues of its mammalian host. Transmitted by the bite of the tsetse (*Glossina* spp.), *T*. *brucei* infects mammals in sub-Saharan Africa. Two subspecies, *T*. *brucei gambiense* and *T*. *b*. *rhodesiense*, infect humans, causing human African trypanosomiasis, a fatal disease when left untreated. Another subspecies, *T*. *b*. *brucei*, infects animals, causing animal African trypanosomiasis, a disease whose effect on domestic livestock poses a huge economic burden to sub-Saharan Africa. The parasite lives extracellularly in the blood and tissues of its mammalian hosts, and VSG is key to long-term infection in this harsh environment. This glycophosphatidylinositol (GPI)-anchored glycoprotein is extremely abundant on the parasite surface, with an estimated 10^7^ copies covering the plasma membrane (Fig 1).

VSG gets the “V” in its name from *T*. *brucei*'s large genomic repertoire of VSG-encoding genes. During an infection, the parasite undergoes antigenic variation in which it “switches” expression of the VSG, drawing from a genomic repertoire of >1,000 VSG-encoding genes (the precise size of this repertoire probably varies between subspecies). In *T*. *b*. *brucei*, about 80% of this repertoire consists of incomplete genes or pseudogenes [1,2]. A VSG mRNA is transcribed from one of ~15 telomeric bloodstream expression sites (BESs), while all other BESs remain transcriptionally silent [3]. Thus, only one VSG covers the parasite surface at any time, except when the parasite is in the process of VSG switching. To change the expressed VSG, transcription can be turned off at one BES and turned on at another (in situ switching), or new VSG genes can be moved into a BES by gene conversion [4]. VSG switching can also occur by telomere exchange, in which VSGs are swapped through recombination between two BESs [4].

## How Does VSG Contribute to Immune Evasion?

It is notable that *T*. *brucei* survives extracellularly within its host while covered by the highly antigenic VSG. Indeed, the parasite takes advantage of the abundance and immunogenicity of its VSG coat to actively evade recognition by the mammalian host. VSG comprises ~95% of the surface proteome [5] and extends ~12–15 nM from the cell membrane [6]. Both by virtue of density and through steric hindrance, VSG is thought to “shield” invariant surface proteins from binding by host antibody (Fig 1). However, there is evidence that immunoglobulin G (IgG) can reach at least partially into the VSG monolayer [7], and modeling suggests that certain invariant surface proteins may reach above the top of the VSG barrier [6]. Thus, some non-VSG proteins might be accessible to host antibody. The absence of an effective immune response to non-variant proteins may be mediated by the abundance and/or immunodominance of VSG or by other unknown mechanisms.

Besides potentially shielding invariant epitopes, the VSG coat functions to selectively remove VSG-specific antibodies from the cell surface, thus effectively dampening the host antibody response [9]. All endocytosis and exocytosis occurs through the flagellar pocket of the parasite, and VSG is continuously endocytosed at this location. The parasite quickly degrades antibody bound to endocytosed VSG while shuttling the VSG, now cleared of bound antibody, back to the surface. Though the precise molecular mechanism that underlies this process is not completely clear, it appears to occur through hydrodynamic-flow–mediated forces, with the bound antibody acting as a “molecular sail.” This means that antibody-bound VSG reaches the flagellar pocket more quickly than bare VSG, and that the bulky multimeric immunoglobulin M (IgM), which is the first antibody isotype produced in response to VSG and the primary isotype responsible for parasite clearance, is cleared more quickly than other isotypes (e.g., dimeric IgG). Thus, at lower antibody concentrations, antibody degradation by *T*. *brucei* can result in evasion of complement-mediated lysis and opsonization.

VSG turnover and antibody degradation only delay clearance by host antibody; at high antibody titers, this turnover is insufficient for evasion of the host’s antibody response. *T*. *brucei* circumvents its eventual recognition by antibody through switching of its VSG coat [10]. During an infection, some parasites within a population will switch their expressed VSG to a new and ideally—at least from the parasite’s point of view—antigenically distinct variant. As the host immune system recognizes a VSG and begins to clear the parasites expressing it, some parasites will have switched to a new variant. This results in characteristic waves of parasitemia in the blood of an infected host as new variants arise and the immune system eliminates them.

## What Are the Dynamics of this Cat-and-Mouse Game?

The characteristic waves of parasitemia during *T*. *brucei* infection were first observed over a hundred years ago, but the precise dynamics of the interaction between VSG and the humoral immune response are still being teased out. VSG switching has long been proposed to be “semi-predictable,” with certain VSGs tending to appear earlier in infection than others [11]. This observed loose order is related to the genomic location of each VSG. BES-associated VSGs, for example (BESs share a great deal of homologous sequence), are almost always detected early in infection.

**Fig 2:**
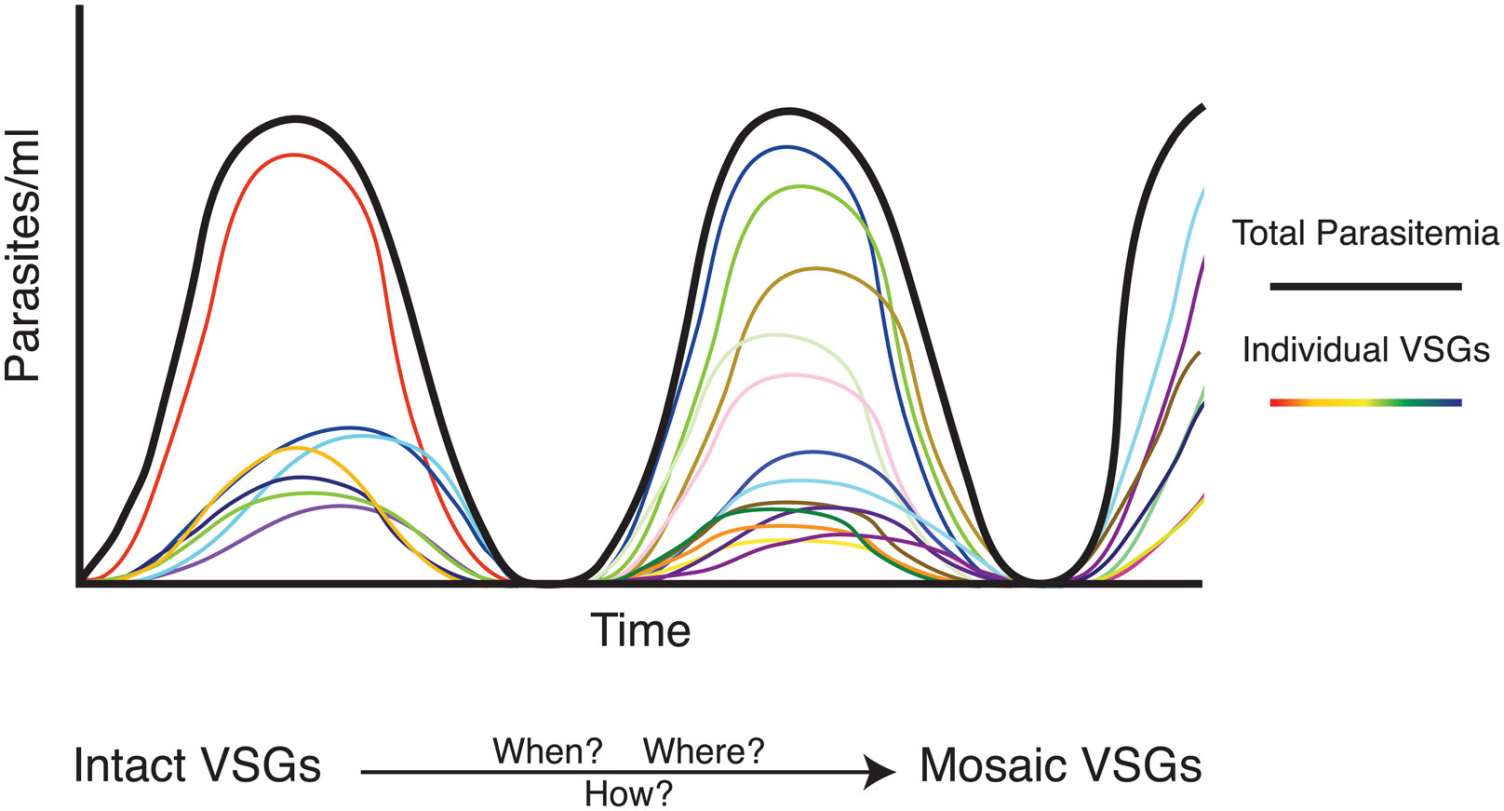
VSG expression dynamics in vivo. The black line represents the total number of parasites at any given point, and the colored lines represent the number of parasites expressing an individual VSG. Recent work has shown a large diversity of VSGs expressed at one time, emphasizing the importance of the formation of mosaic VSGs, which probably predominate later in infection. Little is known, however, about the mechanisms by which these mosaic variants form.

Regardless of the order of VSG expression, the rate at which switching occurs is likely to have a substantial effect on infection dynamics. In theory, the parasite should switch at a rate sufficient for evasion of the immune response but not so fast that it exhausts the genomic repertoire of VSGs. Two recent studies, however, show significant VSG diversity during infection, an observation contrary to a very low rate of switching that would be just sufficient for immune evasion (Fig 2) [12,13]. It is estimated that the trypanosome population at any point during infection can express up to 100 VSGs, and as many as 83 have been definitively measured. This diversity may compensate for frequent VSG failure: although many variants can be detected at any time, only about half establish within the population. Additionally, the formation of so-called “mosaic” VSGs—novel variants that form through gene conversion events within VSG sequences—is likely to be inefficient, as these events probably produce many non-functional or cross-reactive VSGs.

Despite these inefficiencies, mosaic VSGs must predominate later in infection due to the number of VSGs expressed early in infection. There are only ~400 “complete” VSGs (those with a complete open reading frame producing a functional VSG) in annotated *T*. *b*. *brucei* genomes, so the parasite must generate new variants after this intact genomic repertoire has been exhausted [14]. Mosaic VSGs could be crucial to maintaining a chronic infection, which can last several months to years in the wild, but little is known about the rate or mechanism of their formation (Fig 2). It is not yet clear whether a predominance of mosaic VSGs affects parasite dynamics in vivo.

Explicit measurements of the rate of VSG switching in African trypanosomes have been variable. Some in vivo studies suggest a rate as high as 10^−3^ switches/population doubling [15], while studies in vitro demonstrate much lower rates in the range of 10^−6^–10^−5^ [16,17]. This variation is likely a function of the different parasite strains used in each experiment, but it is also possible that the rate of switching is variable and modulated in response to some environmental factor, perhaps one that is absent in vitro. Indeed, one study found that the rate of switching in the same strain of parasites increases upon transmission through a fly [18], highlighting an important caveat to many studies of *T*. *brucei* antigenic variation: most work has been performed using mouse models and lab-adapted *T*. *b*. *brucei* strains. Thus, it remains unclear how these findings translate to the context of natural infections. It is also unclear exactly how other factors, such as quorum sensing [19] or the movement of parasites between tissues [20], affect the rate of switching or the shape and frequency of the parasitemic waves observed during infection.

## How Does the Host Recognize VSG?

On the host side of this arms race, IgM appears to be the primary antibody isotype responsible for parasite recognition and clearance [21]. This is likely because IgM and its splice variant, immunoglobulin D (IgD), are the first antibody isotypes expressed by B cells. The population of parasites expressing a given VSG is usually cleared within a week after appearance, before IgG can be generated. IgM is not, however, required for the maintenance of a chronic infection, as demonstrated in [21] using IgM^-/-^ mice. But IgM^-/-^ mice still express IgD, an isotype whose function in the immune response is an enduring mystery [22], as well as VSG-specific IgG2a and IgG3, leaving the precise role of IgM in parasite recognition and clearance a critical question.

Though the antibody–VSG molecular interface represents the primary host–parasite interaction in a trypanosome infection, little is known about how VSGs and their cognate antibodies interact. For example, the role of the likely immunogenic glycosylation of VSG in antibody recognition of *T*. *brucei* has not yet been explored. Attempts to decipher epitopes recognized by host antibodies have resulted in more confusion than clarity: epitopes have been described that appear more membrane-proximal than expected, quite close to the buried end of the VSG, rather than at the exposed and variable N-terminus [6]. This could be true, as there is evidence that IgG can access more internal VSG epitopes that are probably inaccessible to IgM [7], but the integrity of the coat is suspect in some experimental situations. If the primary isotype recognizing VSG in a natural infection is IgM, however, VSG-internal epitopes may not be relevant to parasite clearance (Fig 1). Therefore, the epitopes recognized by the host are likely to be determined by both the antibody isotype dominating the B cell response as well as the specific antibody–VSG interaction. As the field continues to grapple with antigenic variation, an understanding of the major VSG epitopes recognized during infection is urgently needed, particularly in the context of VSG switching that results in mosaic formation.

## What’s Next for VSGs?

Many questions remain to be answered about the host–parasite interface in *T*. *brucei* infection:

- What determines the rate of switching? Can environmental factors influence this rate?
- Is genomic location the primary determinant of VSG switching order?
- How do mosaic VSGs form, and how often?
- What epitopes are targeted by host antibody?
- How does antibody bind to VSG?
- What is the role of antigen cross-reactivity in the dynamics of infection?

Recent technical advances, particularly in high-throughput sequencing, are allowing key questions about the dynamic interaction of *T*. *brucei* and its host to be answered more precisely now than ever before.
